# Supplementary material for: MetaRibo-Seq measures translation in microbiomes
Source: Nat Commun. 2020 Jun 29;11:3268. doi: 10.1038/s41467-020-17081-z (PMC7324362; doi:10.1038/s41467-020-17081-z)
Supplement: Supplementary file 10 — Supplementary Data 7 [file 41467_2020_17081_MOESM10_ESM.zip › File2/Confidence_High_Taxonomy/155160_out.krona.html]

Javascript must be enabled to view this page.

members
magnitude
magnitudeUnassigned
count
unassigned
taxon
rank

155160\_out

340

2
340
superkingdom

976
phylum
340

200643
340
class

171549
340
order

815
family
335

32
816

SRS015065\_contig\_number\_19509SRS015264\_contig\_number\_contig-100\_1849.110300SRS015431\_contig\_number\_19082SRS016095\_contig\_number\_contig-100\_3201.143481SRS016753\_contig\_number\_5805SRS018427\_contig\_number\_30020SRS018817\_contig\_number\_23179SRS019030\_contig\_number\_contig-100\_1778.104735SRS019381\_contig\_number\_12056SRS020394\_contig\_number\_14408SRS020622\_contig\_number\_2523SRS024331\_contig\_number\_34541SRS024625\_contig\_number\_18578SRS049896\_contig\_number\_8105SRS049900\_contig\_number\_9340SRS050941\_contig\_number\_16155SRS065176\_contig\_number\_3431SRS077454\_contig\_number\_6224SRS077730\_contig\_number\_15811SRS103987\_contig\_number\_14373SRS1041090\_contig\_number\_4958SRS1041137\_contig\_number\_29457SRS104975\_contig\_number\_20143SRS143722\_contig\_number\_contig-100\_1507.90818SRS143876\_contig\_number\_26912SRS144603\_contig\_number\_15654SRS146764\_contig\_number\_8620SRS146812\_contig\_number\_50439SRS147346\_contig\_number\_33108SRS893253\_contig\_number\_1601SRS893279\_contig\_number\_15069SRS893288\_contig\_number\_3965
genus
335

818
3
species

SRS077849\_contig\_number\_12173SRS097920\_contig\_number\_19805SRS1055022\_contig\_number\_3099

3

SRS012902\_contig\_number\_169SRS104485\_contig\_number\_36SRS147557\_contig\_number\_29050
species
817

species

SRS016203\_contig\_number\_contig-100\_501.82854SRS049446\_contig\_number\_13544SRS062427\_contig\_number\_8002SRS074670\_contig\_number\_2974SRS075398\_contig\_number\_2844SRS149181\_contig\_number\_29993SRS893172\_contig\_number\_9405SRS893358\_contig\_number\_10698
8
671267

47678
9

SRS014683\_contig\_number\_13849SRS018623\_contig\_number\_6714SRS019161\_contig\_number\_8346SRS019397\_contig\_number\_8683SRS045826\_contig\_number\_3013SRS065397\_contig\_number\_2955SRS143523\_contig\_number\_23449SRS146813\_contig\_number\_4091SRS148196\_contig\_number\_36139
species

1

SRS075878\_contig\_number\_19112
species
1897052

2292944
2
species

SRS020233\_contig\_number\_25449SRS1041157\_contig\_number\_5440

species

SRS012969\_contig\_number\_31961SRS019685\_contig\_number\_1499
2
371601

species

SRS014459\_contig\_number\_27896SRS016056\_contig\_number\_1013SRS042966\_contig\_number\_11608SRS050752\_contig\_number\_1602SRS076876\_contig\_number\_61SRS098881\_contig\_number\_8262SRS1055076\_contig\_number\_3052SRS893378\_contig\_number\_738
8
46506

310297
2

SRS015663\_contig\_number\_13171SRS105082\_contig\_number\_18873
species

1263056
1

SRS1055095\_contig\_number\_2022
species

376805
3
species

SRS017247\_contig\_number\_1186SRS054059\_contig\_number\_5187SRS063813\_contig\_number\_2348

821
species

SRS011084\_contig\_number\_14340SRS011239\_contig\_number\_15597SRS011302\_contig\_number\_12401SRS011405\_contig\_number\_14945SRS012849\_contig\_number\_22583SRS013158\_contig\_number\_1274SRS013382\_contig\_number\_363SRS013521\_contig\_number\_628SRS013638\_contig\_number\_3429SRS013687\_contig\_number\_3908SRS013965\_contig\_number\_34599SRS014287\_contig\_number\_2894SRS014313\_contig\_number\_4478SRS014412\_contig\_number\_18006SRS014534\_contig\_number\_447SRS014736\_contig\_number\_4842SRS014855\_contig\_number\_15732SRS014948\_contig\_number\_17039SRS014979\_contig\_number\_17890SRS015190\_contig\_number\_6822SRS015217\_contig\_number\_23559SRS015369\_contig\_number\_588SRS015486\_contig\_number\_461SRS015578\_contig\_number\_36968SRS015694\_contig\_number\_4811SRS015854\_contig\_number\_18782SRS015960\_contig\_number\_12839SRS016132\_contig\_number\_4411SRS016335\_contig\_number\_25978SRS016495\_contig\_number\_18070SRS016629\_contig\_number\_3681SRS016840\_contig\_number\_contig-100\_23.37180SRS016954\_contig\_number\_19938SRS017103\_contig\_number\_14305SRS017191\_contig\_number\_18814SRS017307\_contig\_number\_30799SRS017375\_contig\_number\_contig-100\_594.18599SRS017433\_contig\_number\_2598SRS017521\_contig\_number\_21448SRS017745\_contig\_number\_2485SRS017859\_contig\_number\_863SRS018351\_contig\_number\_9104SRS018836\_contig\_number\_17202SRS018888\_contig\_number\_9767SRS018936\_contig\_number\_4069SRS019068\_contig\_number\_38202SRS019178\_contig\_number\_1505SRS019267\_contig\_number\_2054SRS019496\_contig\_number\_11410SRS019601\_contig\_number\_contig-100\_1177.158150SRS019693\_contig\_number\_9542SRS019808\_contig\_number\_20855SRS019910\_contig\_number\_11048SRS019968\_contig\_number\_14712SRS020508\_contig\_number\_112SRS020869\_contig\_number\_5819SRS021153\_contig\_number\_1025SRS021219\_contig\_number\_7257SRS021484\_contig\_number\_804SRS021948\_contig\_number\_10791SRS022713\_contig\_number\_851SRS023829\_contig\_number\_686SRS023914\_contig\_number\_7168SRS023971\_contig\_number\_675SRS024009\_contig\_number\_2925SRS024075\_contig\_number\_3026SRS024492\_contig\_number\_16171SRS043411\_contig\_number\_16291SRS043667\_contig\_number\_4252SRS045004\_contig\_number\_14326SRS045244\_contig\_number\_2492SRS045645\_contig\_number\_15592SRS045739\_contig\_number\_3301SRS046369\_contig\_number\_2434SRS046502\_contig\_number\_13664SRS046810\_contig\_number\_contig-100\_39.40SRS047433\_contig\_number\_12989SRS048164\_contig\_number\_13834SRS048870\_contig\_number\_4857SRS049402\_contig\_number\_1954SRS049712\_contig\_number\_9139SRS049773\_contig\_number\_31441SRS049959\_contig\_number\_30985SRS049995\_contig\_number\_14810SRS050026\_contig\_number\_3933SRS050422\_contig\_number\_23969SRS050520\_contig\_number\_113SRS050801\_contig\_number\_3852SRS050974\_contig\_number\_contig-100\_77.24116SRS050998\_contig\_number\_16477SRS051610\_contig\_number\_8690SRS051882\_contig\_number\_13546SRS052697\_contig\_number\_14607SRS053087\_contig\_number\_590SRS053214\_contig\_number\_1290SRS053335\_contig\_number\_17277SRS053356\_contig\_number\_47020SRS053398\_contig\_number\_12110SRS054352\_contig\_number\_18676SRS054905\_contig\_number\_4713SRS055017\_contig\_number\_15312SRS055966\_contig\_number\_75SRS056273\_contig\_number\_28561SRS056537\_contig\_number\_61401SRS057478\_contig\_number\_1973SRS057480\_contig\_number\_1825SRS058268\_contig\_number\_46SRS058723\_contig\_number\_1159SRS062654\_contig\_number\_13454SRS062701\_contig\_number\_4884SRS063127\_contig\_number\_871SRS063190\_contig\_number\_12787SRS063370\_contig\_number\_683SRS063928\_contig\_number\_45SRS064557\_contig\_number\_2081SRS064645\_contig\_number\_316SRS064973\_contig\_number\_29SRS065504\_contig\_number\_23487SRS074586\_contig\_number\_contig-100\_553.55527SRS074964\_contig\_number\_2010SRS075078\_contig\_number\_16516SRS075716\_contig\_number\_3914SRS075773\_contig\_number\_contig-100\_2431.196695SRS075821\_contig\_number\_13292SRS075984\_contig\_number\_1976SRS076756\_contig\_number\_19275SRS076976\_contig\_number\_18294SRS077194\_contig\_number\_12426SRS077231\_contig\_number\_9825SRS077294\_contig\_number\_15234SRS077335\_contig\_number\_1233SRS077502\_contig\_number\_16340SRS077552\_contig\_number\_12486SRS077589\_contig\_number\_836SRS077641\_contig\_number\_19889SRS077991\_contig\_number\_contig-100\_191.24098SRS078176\_contig\_number\_contig-100\_1953.121886SRS078242\_contig\_number\_19408SRS078665\_contig\_number\_906SRS097889\_contig\_number\_18047SRS097958\_contig\_number\_11442SRS098061\_contig\_number\_contig-100\_350.85836SRS098073\_contig\_number\_4437SRS098571\_contig\_number\_14630SRS098644\_contig\_number\_23818SRS098827\_contig\_number\_1052SRS100021\_contig\_number\_1932SRS100907\_contig\_number\_3225SRS101376\_contig\_number\_17984SRS101433\_contig\_number\_10975SRS104036\_contig\_number\_contig-100\_377.62371SRS104084\_contig\_number\_1862SRS1041031\_contig\_number\_27574SRS1041032\_contig\_number\_17468SRS1041036\_contig\_number\_2403SRS1041037\_contig\_number\_2620SRS1041091\_contig\_number\_9208SRS1041092\_contig\_number\_9967SRS1041095\_contig\_number\_948SRS1041116\_contig\_number\_contig-100\_40.56301SRS1041118\_contig\_number\_11172SRS1041130\_contig\_number\_591SRS1041134\_contig\_number\_4918SRS1041139\_contig\_number\_3029SRS1041140\_contig\_number\_21826SRS1041141\_contig\_number\_1973SRS1041142\_contig\_number\_7371SRS1041143\_contig\_number\_6990SRS1041144\_contig\_number\_7050SRS1041147\_contig\_number\_2217SRS1041164\_contig\_number\_5341SRS104327\_contig\_number\_3500SRS104400\_contig\_number\_23213SRS104693\_contig\_number\_8629SRS104912\_contig\_number\_19356SRS105153\_contig\_number\_14472SRS1054716\_contig\_number\_15105SRS1054928\_contig\_number\_5692SRS1055038\_contig\_number\_1438SRS1055049\_contig\_number\_11564SRS1055056\_contig\_number\_13127SRS1055083\_contig\_number\_9716SRS140492\_contig\_number\_13301SRS142503\_contig\_number\_27001SRS142542\_contig\_number\_12587SRS142781\_contig\_number\_3801SRS142890\_contig\_number\_26463SRS142923\_contig\_number\_7584SRS142980\_contig\_number\_5615SRS143070\_contig\_number\_3690SRS143085\_contig\_number\_5227SRS143148\_contig\_number\_14567SRS143181\_contig\_number\_11840SRS143372\_contig\_number\_2533SRS143417\_contig\_number\_29045SRS143780\_contig\_number\_1251SRS143895\_contig\_number\_39611SRS143991\_contig\_number\_15028SRS144135\_contig\_number\_24334SRS144183\_contig\_number\_40880SRS144362\_contig\_number\_2415SRS144506\_contig\_number\_23611SRS144537\_contig\_number\_40112SRS144714\_contig\_number\_14422SRS144753\_contig\_number\_11897SRS145308\_contig\_number\_1153SRS147022\_contig\_number\_256SRS147039\_contig\_number\_2698SRS147271\_contig\_number\_26532SRS147377\_contig\_number\_4955SRS147425\_contig\_number\_1098SRS147614\_contig\_number\_8612SRS147766\_contig\_number\_7869SRS147977\_contig\_number\_8732SRS148091\_contig\_number\_9632SRS148253\_contig\_number\_8890SRS148721\_contig\_number\_45609SRS148784\_contig\_number\_14061SRS148817\_contig\_number\_11976SRS148970\_contig\_number\_1099SRS149075\_contig\_number\_4993SRS149244\_contig\_number\_5368SRS149325\_contig\_number\_10249SRS892882\_contig\_number\_15486SRS893170\_contig\_number\_5535SRS893187\_contig\_number\_11529SRS893230\_contig\_number\_8171SRS893252\_contig\_number\_13309SRS893256\_contig\_number\_1211SRS893259\_contig\_number\_6383SRS893285\_contig\_number\_1386SRS893327\_contig\_number\_4450SRS893341\_contig\_number\_11739SRS893342\_contig\_number\_8480SRS893366\_contig\_number\_24366SRS893369\_contig\_number\_2176SRS893373\_contig\_number\_24259SRS893380\_contig\_number\_761SRS971275\_contig\_number\_17151
239

204516
species

SRS016989\_contig\_number\_1379SRS022609\_contig\_number\_21537SRS052027\_contig\_number\_8834SRS1041133\_contig\_number\_27815
4

1262737
species

SRS893382\_contig\_number\_347
1

2302926

SRS063518\_contig\_number\_35310
species
1


SRS016897\_contig\_number\_78SRS893270\_contig\_number\_22963
species
2
820

species

SRS023526\_contig\_number\_13853
1
291645

457387

SRS893295\_contig\_number\_13472
species
1

2
species

SRS050299\_contig\_number\_2221SRS150029\_contig\_number\_16944
357276

1896973

SRS1041129\_contig\_number\_2103
species
1

species

SRS042284\_contig\_number\_4450
1
28116

2292001
species

SRS893383\_contig\_number\_20428
1


SRS052576\_contig\_number\_107
species
1
1739298

species

SRS019445\_contig\_number\_12947SRS143342\_contig\_number\_205
2
1896974


SRS142599\_contig\_number\_30089
species
1
329854

310298
1
species

SRS051031\_contig\_number\_30450

338188
1
species

SRS140513\_contig\_number\_8661

species

SRS063040\_contig\_number\_2170
1
376806

1
family
171550

1
genus
239759

1

SRS020328\_contig\_number\_11410
species
28117

1853231
family
1

283168
genus
1

28118
1
species

SRS148424\_contig\_number\_21553

family
1
2005525

375288
1
genus


SRS047014\_contig\_number\_23116
species
1
46503

family
2
171552

577309
genus
1

454154
1
species

SRS1041138\_contig\_number\_7217

1
genus
838

165179

SRS144297\_contig\_number\_4284
species
1
